# Osseointegrated Supported Prosthesis and Interdisciplinary Approach for Prosthodontic Rehabilitation of a Young Patient with Ectodermal Dysplasia

**DOI:** 10.1155/2013/963191

**Published:** 2013-09-18

**Authors:** Karthik M. Sadashiva, N. Sridhar Shetty, Rakshith Hegde, Mallika M. Karthik

**Affiliations:** ^1^Department of Dental and Prosthetic Surgery, Tata Memorial Hospital, Dr. E. Borges Marg, Parel, Mumbai 400012, India; ^2^Department of Prosthodontics, A. B. Shetty Dental College, Deralakatte, Mangalore 575018, India; ^3^Department of Prosthodontics, Guardian College of Dental Sciences & Research Centre, Jambhulgaon Road, Chikhloli, Ambarnath W., Thane District 421503, India

## Abstract

Anhidrotic ectodermal dysplasia is a triad of hypodontia or anodontia, hypotrichosis, and hypohydrosis, associated with other problems that result from the defective development of structures of ectodermal origin (Freire-Maia, Pinheiro (1988)). Early and extensive dental treatment is needed keeping in mind the effect on the craniofacial growth. Due to rapid growth of the jaws, the patients are rehabilitated using removable prostheses (Tarjan et al. (2005)). Hence for a young patient in this case report, the placement of endosseous osseointegrated implants was delayed till adulthood. Finally a definitive fixed tooth-supported and osseointegrated implant supported fixed partial denture therapy was used to rehabilitate the patient satisfactorily after she had completed her growth (Sweeney et al. (2005)). A review of the current literature relevant to several aspects of syndromic hypodontia, patient selection, and implant planning is discussed.

## 1. Introduction

Ectodermal dysplasia syndrome was first described in the medical literature by Thurnam, who reported two typical patients as carriers of an x-linked recessive disorder, in 1848 [1]. It was reported that, especially in mild forms of ED, the most common complaint of childhood and adolescence is the concern about the dental abnormalities and facial appearance resulting in a state of depression when they compare themselves with other children [2]. In this case report the physical development of the 14-year-old female patient having anhidrotic ectodermal dysplasia was incomplete. She required the support in coping with issues of attractiveness during the formative years of childhood and adolescence. Transitional removable partial and complete dentures were planned for her oral rehabilitation, which allowed adjustments. Osseointegrated implants and permanent prosthodontic treatment were carried out when she turned 21 years of age.

## 2. Case Report

**Figure 1:**
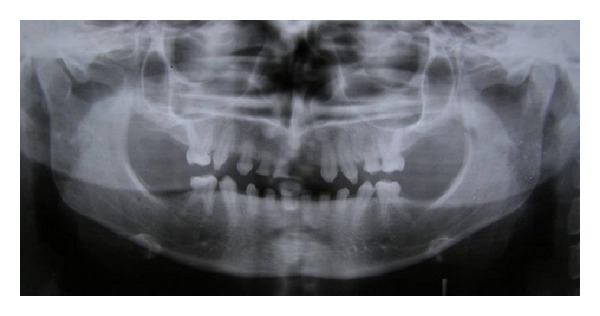
Preoperative radiograph showing overretained deciduous teeth and a few permanent teeth.

**Figure 2:**
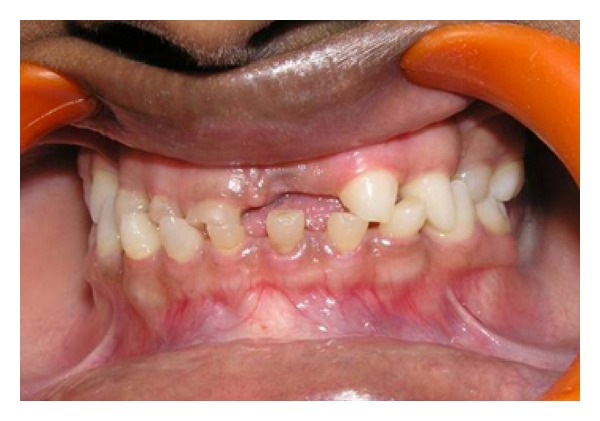
Severely attritted mixed dentition resulting in a collapsed bite.

**Figure 3:**
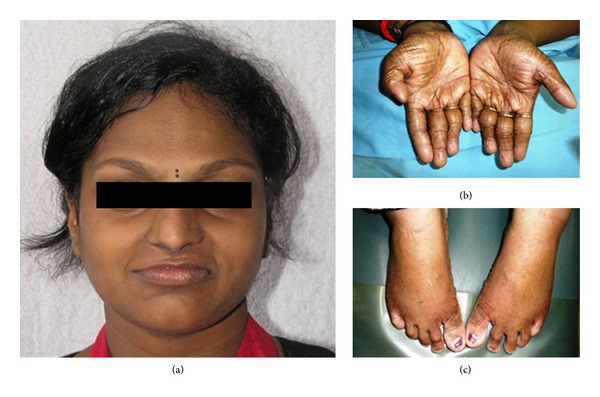
The patient exhibiting prominent and protuberant lips, abnormal hair (trichodysplasia) (head and eyebrows) (a), dry wrinkled skin of the palm (b), and feet with abnormal nails (onychodysplasia) (c).

A 14-year-old Asian female presented with having some phenotypic expressions with mild symptoms with prominent and protuberant lips, abnormal hair (trichodysplasia) (head and eyebrows), skin of hands, palm, and feetdry, wrinkled, and scaly with abnormal nails (onychodysplasia) (Figure 3), and hyperpyrexia due to reduced number of sweat glands (dyshidrosis). She complained of esthetic impairment, inability to eat, and difficulty in speech due to several mobile, malformed, hypoplastic, and missing teeth. Radiographic examination showed over retained deciduous teeth, 51, 52, 53, 64, 71, 72, 73, 81, 82 and 83, and few permanent teeth, 15, 16, 23, 24, 26, 35, 36, 45, and 46 (Figure 1). On clinical examination intraorally the deciduous teeth (51, 52, 53, 64, 71, 72, 73, 81, 82, and 83) were showing grade I mobility and severe attrition resulting in reduced lower facial height and a collapsed bite (Figure 2). The oral manifestations of the patient that affected the dental treatment were oligodontia, hypoplastic teeth, overretention of deciduous teeth, alterations of teeth shape, and missing permanent tooth buds for most of the teeth [3]. 

**Figure 4:**
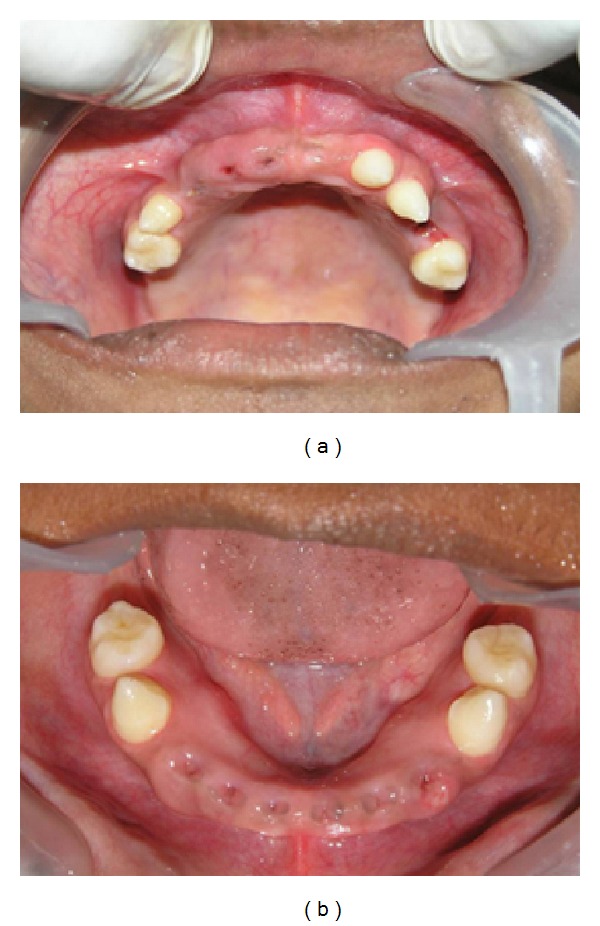
Maxillary and mandibular arch after extraction of mobile deciduous teeth.

The advantages of existing teeth with regard to retention, stability, function, and the phonetics of the dentures were considered and were preserved as much as possible. Only her deciduous teeth were extracted [4]. Since the patient retained most of her deciduous teeth till the age of 14 there was no significant deficiency in the development of the alveolar process (Figure 4). The patient was apprehensive about the treatment and agreed for only interim removable partial dentures to begin with. Followup was done every month, and few adjustments were performed in the interim removable partial dentures accordingly.

**Figure 5:**
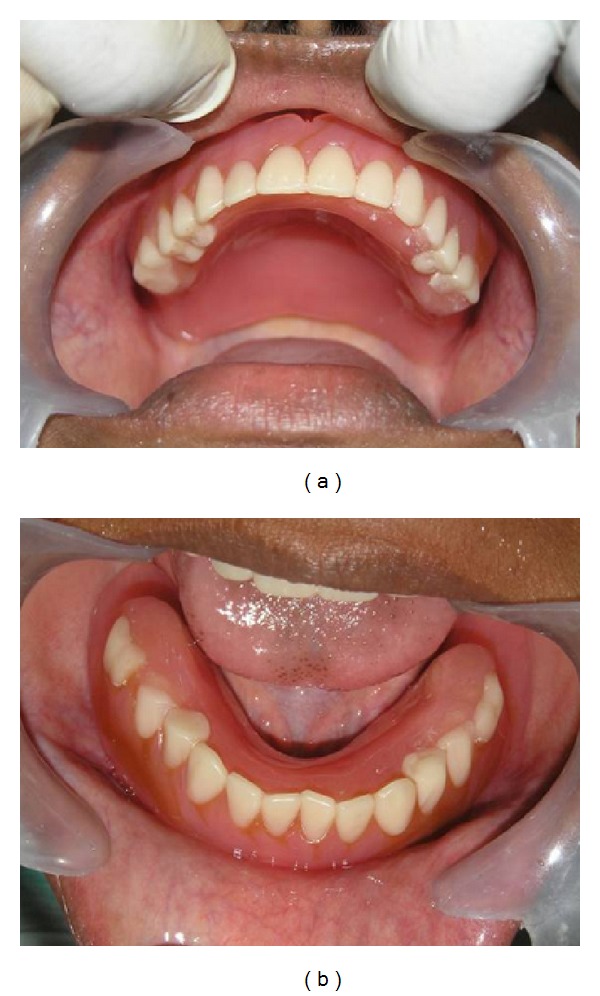
Conventional overdenture prosthesis inserted in the patient's mouth.

*After 1 Year, Patient's Age: 15 yrs.* After using interim removable partial dentures for 1 year she complained of poor retention of the prostheses. A conventional overdenture prosthesis was then planned and fabricated for the patient without preparing the existing permanent teeth (Figure 5). Followup was carried out every month, and few adjustments were performed in the overdentures accordingly.

**Figure 6:**
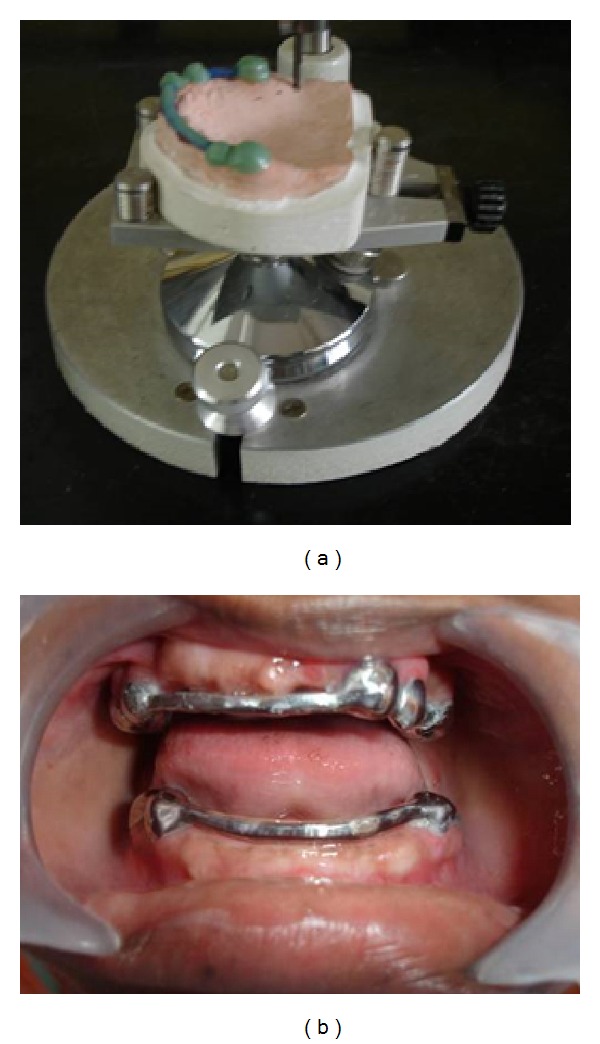
Milling of wax pattern on the cast (a) and milled metallic framework cemented to the teeth intraorally (b).

*After 3 Years, Patient's Age: 18 yrs.* The patient returned after 3 yrs with complaints of poor retention with the overdenture prosthesis which had been relined over these 3 years periodically and needed complete replacement. Once again after careful evaluation, a tooth and bar supported overdenture prosthesis was planned to enhance the retention. The patient agreed for minimal tooth preparation of the remaining permanent teeth to receive metal copings (Figure 6).

*After 3 Years, Patient's Age: 21 yrs.* The patient at the age of 21 yrs desired to have fixed teeth with no palatal coverage. She complained of discomfort due to food lodgment under the bar attachment and required use of meticulous oral hygiene measures to keep the mucosa clean under the bar attachment. Fixed osseointegrated implant supported prosthesis was planned for the edentulous spans. Patient had adequate lip support, an average smile line, and an average length of the upper lip. Bone mapping and bone scans were done to assess the quality and quantity of available bone. Although the facial profile was Angle's class III with a collapsed lower facial height, the intermaxillary relationship was an Angle's class I.

**Figure 7:**
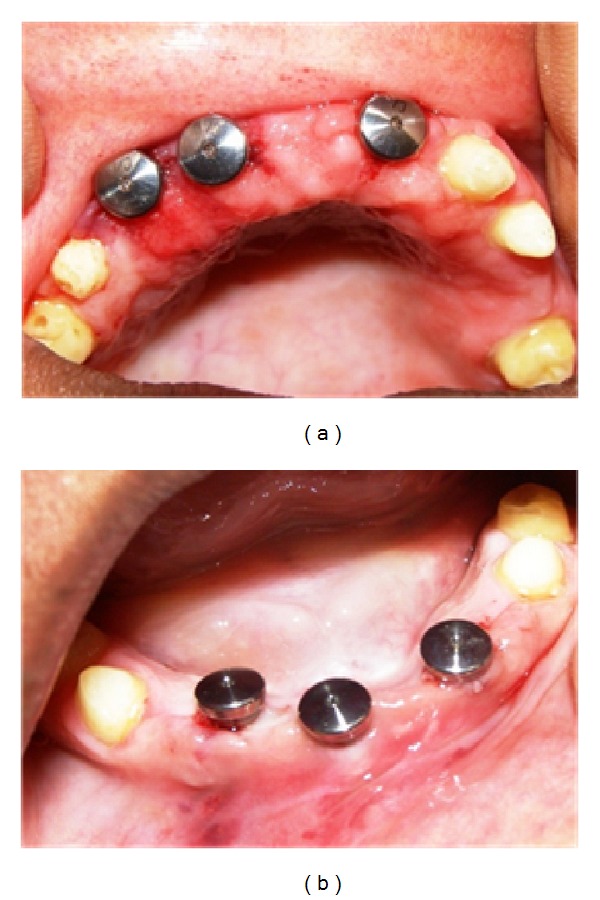
Implants in maxillary (12, 13, and 21 regions) (a) and mandibular (41, 43, and 32 regions) (b) with attached gingival formers.

The implant positions were planned on articulated cast models and transferred into surgery using a surgical stent. Endosseous implants were placed in the maxillary 12, 13, and 21 regions (A-11, Ankylos, Dentsply India Pvt. Ltd.) and mandibular 41, 43, and 32 regions (A-11, Ankylos, Dentsply) (Figure 7).

The natural teeth were temporized with new acrylic copings, and the overdenture prosthesis was relined with tissue conditioners and inserted in the patient's mouth. Implants were allowed to osseointegrate for 3 months. Second stage surgery was then done, and gingival formers were placed.

**Figure 8:**
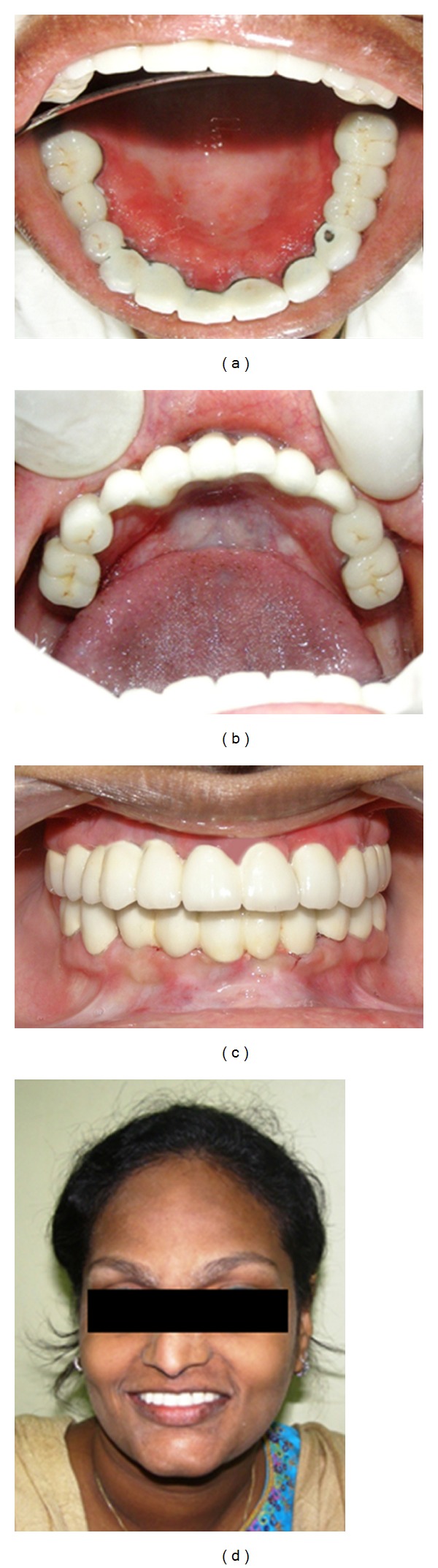
Occlusal view of the prosthesis, maxillary arch (mirror image) (a), mandibular arch (b) (mirror image), in occlusion (c), and patient's smile (d).

**Figure 9:**
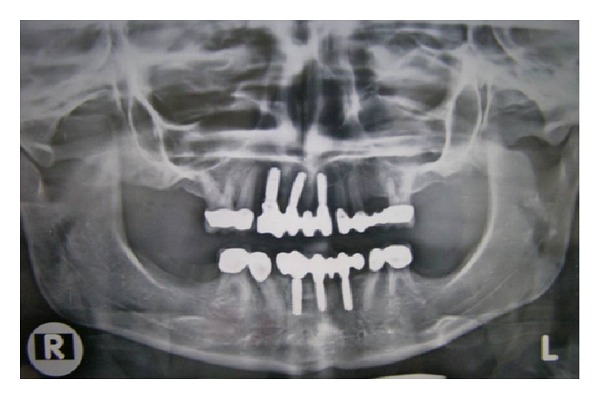
Radiograph taken after 6 months of cementation of the prosthesis.

Permanent crowns and fixed partial dentures were fabricated on the natural teeth. The fabrication of implant supported prosthesis was initiated after 14 days of the second stage surgery. Osseointegrated supported fixed prosthesis was cemented in occlusion (Figure 8). The patient was instructed to maintain oral hygiene and return periodically for followup visits. The patient was satisfied with her prosthesis both aesthetically and functionally. A radiograph was taken after 6 months of cementation of the prosthesis (Figure 9).

## 3. Discussion

Reports in the literature describe placement of dental implants as early as 3 years of age in the mandible [5]. But in the following years the mandibular rehabilitation becomes increasingly difficult due to growth of the mandible and deficient height of the alveolar processes [6]. In the mandible, the transverse skeletal or alveolodental changes are less dramatic as in the maxilla. The insertion of dental implants in children or adolescents before completion of craniofacial growth is said to have imitated the effects of ankylosed teeth [7]. When placed in alignment with adjacent teeth, the implants did not participate in growth processes, resulting in an infraocclusion and multidimensional dislocation when compared with the developing teeth. The adjacent tooth germs exhibited morphologic changes and disorders of eruption [8]. The insertion of implants in the growing maxilla should be avoided until early adulthood as fixed implant constructions crossing the midpalatal suture will result in lack of growth [9]. In the nearly anodontic child, however, these problems can be neglected [10].

It is advisable to delay the prosthetic rehabilitation till the age of 5 where only conventional removable dentures made of heat-cured acrylic resin can be fabricated for both the upper and lower jaws and accepted by the child. The necessary changes can be easily accommodated in these prostheses or they can be easily relined or remade as the person grows [11, 12]. The removable interim prosthodontic treatment, planned over a span of seven years before the placements of endosseous implants, gained the patient's confidence in the treatment plan, and an improvement was observed in the patient's psychosocial behavior.

## 4. Summary and Conclusion

This report describes a potential routine approach to restoring the appearance, function, and psyche of a growing girl with ectodermal dysplasia. A conventional prothetic approach was used to gather functional and esthetic information to aid in the design of the final prosthesis and to allow as much growth as possible before initiating the implant-assisted phase of treatment. The status of skeletal growth, the degree of hypodontia, the status of the existing dentition, and the dental compliance and extension of related psychosocial stress of the patient were taken into account when determining the optimal time point of implant insertion.
